# A Review of Current and Emerging Therapeutic Options for Erectile Dysfunction

**DOI:** 10.3390/medsci7090091

**Published:** 2019-08-29

**Authors:** Eric Chung

**Affiliations:** 1 AndroUrology Centre, Brisbane 4000, Queensland, Australia; 2 University of Queensland, Princess Alexandra Hospital, Brisbane 4102, Queensland, Australia; 3 Macquarie University Hospital, Sydney 2109, New South Wales, Australia

**Keywords:** erectile dysfunction, phosphodiesterase type 5, intracavernosal injection, penile prosthesis implant, low-intensity shock wave, stem cell, gene therapy, platelet rich plasma, microvascular stent, penile transplant

## Abstract

Contemporary treatment algorithms for erectile dysfunction (ED) involve the use of medical therapies such as phosphodiesterase type 5 (PDE5) inhibitors and intracavernosal injection therapy of vasoactive agents, as well as vacuum erection devices and penile prosthesis implants in medically refractory cases. However, the current therapeutic options only address the symptoms of ED and not the underlying pathogenesis that results in ED. Newer and novel ED therapies aspire to reverse ED conditions by preventing cavernosal fibrosis, promoting endothelial revascularization and modulating various neuro-hormonal pathways. Regenerative therapeutic strategies such as low-intensity shock wave, gene and cellular-based therapies, and penile transplants are designed to improve penile hemodynamics and revitalize the cavernosal smooth muscle to mitigate and/or reverse underlying ED. This state-of-art article evaluates current and emerging therapeutic options for ED.

## 1. Introduction

While the introduction of oral phosphodiesterase type 5 (PDE5) inhibitors revolutionized the management of erectile dysfunction (ED), they are not always effective since the development and progression of ED is frequently attributable to both psychogenic factors and physiological alterations in various neural, vascular, hormonal and endothelial functions. Recent epidemiological studies have highlighted the correlation between ED and underlying cardiovascular and metabolic risk factors [1], and international guidelines advocate optimization of underlying medical comorbidities as the first line therapy for men presenting with ED [2,3].

In recent years, there have been significant advances made in the field of sexual medicine in terms of our understanding of the underlying molecular biology and neuro-humoral mechanisms governing male sexual function. In contrast to existing therapeutic approaches, newer and innovative ED therapies aspire to prevent underlying cavernosal fibrosis, promote endothelial revascularization and modulate the neuro-hormonal pathway with angiogenic and tissue growth factors (see Table 1). Gene and cellular-based therapies are designed to act on the molecular level to improve specific cellular and enzymatic functions to mitigate and/or reverse underlying ED. The following article evaluates the current standard treatment strategies as well as emerging novel and innovative therapeutic options in ED.

## 2. Current Standard Erectile Dysfunction Therapy

### 2.1. Oral Phosphodiesterase Type 5 Inhibitor (PDE5i) Therapy

The clinical efficacy and safety profile of PDE5i has been reported by numerous high-quality, well-designed, blinded, randomized controlled trials comparing PDE5i both to placebo and to other PDE5i drugs [3,4,5,6]. Overall, oral PDE5i in recommended doses is an effective medical therapy for ED with an excellent safety profile and is generally well tolerated. The underlying causative factor for ED such as radical prostatectomy [7] or radiation [8] and the presence of significant medical comorbidities with diabetes [9,10] can affect the success rate of PDE5i. Next-generation PDE5i drugs including avanafil (Stendra^®^), mirodenafil (Mvix^®^), lodenafil (Helleva^®^), and udenafil (Zydena^®^) work in a similar manner to increase nitric oxide (NO) concentration and have relatively similar side effect profiles.

It is important to note that PDE5i only works with sexual stimulation, and despite the initial success in 65–70% of patients, 30–40% do not respond to PDE5i alone, so alternative strategies must be considered to enhance the response rate [11]. Realistic expectations should be set, and patients should be encouraged to give the medications a chance to work and be aware of the dietary restrictions and latency time between ingestion and drug effect. In some patients with chronic ED, additional exposure of PDE5i drugs over several occasions might be required before a satisfactory response is achieved. Awareness of the patient and partner’s sexual script will often be useful in determining both the choice of medication and the dosing strategy used [12].

### 2.2. Intracavernosal Injection

Intracavernosal vasoactive drug injection (ICI) therapy is an effective alternative with minimal systemic side effects compared to oral ED therapy [13]. Common vasoactive agents are prostaglandin E1 (PGE1), which stimulates cyclic adenosine monophosphate (cAMP); papaverine, a non-selective PDE5i; and phentolamine, which is a non-selective alpha-adrenergic antagonist that inhibits smooth muscle contraction. PGE1 can be used as a monotherapy or in combination with other vasoactive agents [3]. More recently aviptadil, a synthetic vasoactive intestinal polypeptide (VIP) that increases the activity of adenosine cyclase, was introduced and is available as a combination of aviptadil/phentolamine (Invicorp©) [14].

Clinical guidelines advocate the use of combination intracavernosal therapy as an alternative to monotherapy due to its more favorable side-effect profile and higher efficacy [3,15]. ICI injections are a moderately invasive therapeutic option and require a degree of manual dexterity, from the patient or partner, with education to learn the mechanics of self-injection. It is recommended that patient counselling is pivotal due to high discontinuation rates and risk of priapism, and education on ICI administration techniques and regular patient follow-up are equally important. Discontinuation rates are typically greatest within 3–6 months of commencement and are usually due to factors such as pain, fibrosis, lack of a sexual partner, loss of spontaneity and anxiety [16].

### 2.3. Topical Drugs

Intraurethral alprostadil, often marketed as the Medicated Urethral System for Erection (MUSE), is a single-use pellet containing alprostadil suspended in polyethylene glycol administered using an applicator. Data from key clinical studies of intraurethral alprostadil show that it has a fast onset and a good safety profile, with no risk of penile priapism, fibrosis (as seen with intracavernosal injection) or other typical systemic effects observed with oral ED drugs [17]. While intraurethral alprostadil has been associated with good clinical efficacy and ease of use, it can cause urethral irritation and dysuria [18].

More recently, topical alprostadil cream has been tested and was reported to have similar clinical efficacy to that of intraurethral application [19]. Using a specialized dispenser, alprostadil cream (Vitaros^®^) appeared to be more effective than the standard administration method and there was no difference in terms of local and systemic side effects [20].

### 2.4. Penile Prosthesis Implant

The advent of the modern penile prosthetic in 1973 offered a “real” treatment option for men with ED for the first time [21]. The non-inflatable or malleable penile prosthesis usually consists of a pair of rods which can be bent upright or downward depending on its use. The most common malleable prostheses are the American Medical Systems (AMS) 600, AMS 650 and Spectra (Boston Scientific, Minnetonka, MN, USA) and Coloplast Genesis (Coloplast, Minneapolis, MN, USA), while others such as the Silimed penile prosthesis (Brazil), Shah penile implant (India), Promedon Tube prosthesis (Argentina) and Zephyr ZSI 100 (Switzerland) have had limited commercial success [22]. The malleable penile prosthesis is an ideal option for those who are physically handicapped with poor hand dexterity or limited finger movement, complain of muscle fatigue (as in neurological disorders), or have limited reach or range of mobility (e.g., spinal patients). While malleable penile prosthesis implants have poor concealment, they are cheaper than inflatable penile prosthesis and have a lower mechanical failure rate due to their minimal components [23].

The Boston Scientific AMS 700 series (the AMS 700 LGX, AMS 700 CX and AMS 700 CXR) and Coloplast Titan device are the two main inflatable prostheses in the market, although the Zephyr ZSI 475 (Zephyr Surgical Implants SRAL, Geneva, Switzerland) is rapidly gaining popularity in certain parts of Europe [22]. The inflatable penile prosthesis implant is considered a superior option to the malleable prosthesis as it produces penile rigidity and flaccidity that closely replicates normal erectile function. Appropriate patient selection and counselling, strict adherence to antimicrobial prophylaxis, and safe surgical practice are paramount to ensure low complication and high patient satisfaction rates. Pre-operative patient counselling is essential to address any unrealistic expectations (including penile length loss associated with ED) and adequately inform patients of potential surgical complications in order to optimize post-operative satisfaction. Innovations in prosthetic technology and advances in surgical techniques have resulted in excellent clinical outcomes, mechanical reliability and high patient satisfaction [24].

### 2.5. Penile Revascularization

From the first penile revascularization surgery performed by Michal in 1972 [25] to microvascular anastomosis refinement by Virag a decade later [26], there have been numerous controversies due to the absence of large prospective and well-controlled studies and the fact that penile prosthesis implants appear to be a more effective solution. Nonetheless, penile revascularization may be offered to younger non-diabetic men (<55 years) with isolated arterial stenosis, usually in the setting of pelvic trauma and without generalized vascular disease. The surgical principle of penile revascularization includes anastomosis of the inferior epigastric artery to the dorsal penile arteries and/or the deep dorsal vein. In contrast, penile venous ligation has largely been abandoned due to poor long-term effect and high complication rates [27].

Extensive vascular evaluation is necessary to define the exact nature of vascular injury and stenosis since standardized criteria for patient selection, follow-up protocol and success definition have yet to be identified. Patients should be counselled regarding the future development of ED and serious complications such as priapism, glans hyperemia, vascular and harvest site complications are not uncommon and can be devastating. Where possible, physiological penile revascularization procedures are preferred and surgical technique should be individualized depending on the pathological findings in each case given that anatomical variations of the penile artery are common.

## 3. Emerging and Innovative Erectile Dysfunction Strategies

### 3.1. Novel Drugs and Drug Delivery Systems

Guanylate cyclase (GC) activators have been shown to increase NO levels and promote vasodilation [28]. In contrast to the classical NO donors, BAY 60-2770 is a soluble GC activator that increases cyclic guanosine monophosphate (cGMP) levels even when there is reduced NO bioavailability (e.g., following pelvic nerve injury) [29] and may offer advantages over conventional PDE5i drugs for treating patients with ED and extensive endothelial damage [30].

Several studies have highlighted important roles for GTPase RhoA and its effector, Rho-associated kinase (Rho-kinase) in the calcium-independent regulation of smooth muscle contraction and penile erection [31]. The literature shows that an elevated RhoA/Rho-kinase activity contributes to the pathogenesis of ED which is common in diabetes, ageing men and hypogonadism [32]. Several RhoA/Rho-kinase inhibitors have been tested in various animal models and showed promising outcomes in experimental models. Chronic treatment with fasudil, an oral RhoA/Rho-kinase inhibitor, prevents the development of both vasculogenic ED and pelvic atherosclerosis [33] while the Rho-kinase inhibitor Y27632 (unlike sildenafil) is largely independent of endothelial NO activity and may be a good option in diabetic and hypertensive patients where endothelial NO activity is usually impaired [34].

During the last decade, innovative drug delivery systems, including orally disintegrating formulations (ODF), have been developed as an alternative to conventional marketed dosage formulations to improve patient convenience and acceptability and enhance compliance [35]. In more recent years, ODFs have been developed with the aim of enhancing clinical efficacy and mechanical strength as well as improving patient compliance and acceptability over conventional solid dosing forms. Both oral dispersible sildenafil [36] and vardenafil [37] have been manufactured with some commercial success.

Solid lipid nanoparticles are lipid carriers that can greatly enhance drug solubility and bioavailability [38] and a variety of lipid vesicles such as ethosomes, transfersomes and penetration enhancing lipid vesicles have been designed with the objective of higher encapsulation rate and higher stability, such as solid lipid nanoparticles and nanostructured lipid nanocarriers [39]. The transdermal bioavailability of a vardenafil nanoethosome film was reported to be two-fold higher than the oral bioavailability from an aqueous suspension [40]. Papaverine with lyotropic liquid crystals has been shown to provide an effective alternative to injectable formulations both in vitro and ex vivo [41].

### 3.2. Low-Intensity Extracorporeal Shockwave Therapy (LIESWT)

The proposed mechanisms of action of low-intensity extracorporeal shock wave therapy (LIESWT) to promote neovascularization are thought to be related to the release of various angiogenic growth factors and activation tissue repair functions such as enhanced macrophage activity, alterations in cellular apoptosis, greater synthesis of cellular proteins and activation as well as enhanced recruitment and subsequent differentiation of stem/progenitor cells [42,43]. There is strong emerging literature to support the use of LIESWT, especially in vasculogenic ED, with many clinical studies reporting encouraging results in the use of LIESWT with improved erectile function, a good safety record and short-term durability [44,45]. The therapeutic efficacy of LIESWT in erectile function recovery appeared to last at least three months during the duration of study and patients with mild/moderate ED reported higher therapeutic efficacy than patients with more severe ED or those with multiple medical comorbidities.

While current clinical studies show that the stimulation effects and therapeutic mechanisms among LIESWT machines are similar, regardless of the physical differences and the treatment template, it remains unknown if one machine is superior to another [46]. Furthermore, there is a need to define the optimal LIESWT treatment protocol including the ideal treatment template, the modality of shock wave energy, the emission frequency and the total energy delivery. More stringent randomized controlled trials with longer-term follow-ups are warranted before LIESWT technology is accepted as the standard of care in ED.

### 3.3. Cellular-Based Therapy

Scientific research into stem cell (SC) therapy has advanced rapidly in the last decade with an increased volume of publications resulting in the clinical translation of SC-based interventions, especially in the field of erectile dysfunction [47]. While a SC is a progenitor cell that has the capability to turn into any cell lineage, the likely mechanism for erectile recovery appears to involve neuronal preservation and cytoprotection by the inhibition of cellular apoptosis [48]. Adipose tissue-derived SC (ADSC) are the most widely used SC type in ED research given that they are easy to obtain from abundant tissue sources [49]. Proposed methods to enhance the therapeutic effects of SC including the use of certain growth factors (e.g., vascular endothelial growth factor (VEGF)-transfected ADSCs) [50], gene manipulation (e.g., the KCNMA1 gene in cellular excitement and neurotransmitter release) [51] and matrixen [52]. Clinical trials in human subjects showed that SC injection is well tolerated and appears to improve erectile function in men following radical prostatectomy [53] and diabetic ED [54] with no significant adverse reactions. In another study, in vitro preconditioning of ADSCs could accelerate functional and structural recovery in vivo, indicating that preconditioning by inhibition of PDE5 may improve ADSC therapy following diabetes-induced ED [55].

On the other hand, platelet-rich plasma (PRP) is an autologous product obtained from whole blood, containing high concentrations of platelet growth factors [56], and provides a fibrin framework over platelets that has the potential to support a regenerative matrix [57]. While existing human clinical trials showed that PRP is well-tolerated, safe, and provides a feasible treatment modality in patients with ED [58,59], there is a need for standardization of PRP processing methods [60] and higher quality randomized controlled trials with larger patient samples and longer-term follow up [61]. Co-administration of mesenchymal SC and platelet lysate in men with ED appears promising and safe [62].

While rapid advances of SC clinical trials are paving the clinical pathway for an emergent new medicine that may one day replace current medical and surgical interventions, there are serious considerations with regards to longer-term clinical efficacy and safety profile as well as the quality of published SC trials. Even when SCs are administered directly, they might not always migrate or differentiate as desired, either of which could create a risk of harm, in addition to compromising the effectiveness of the treatment. Safety concerns such as the risk of malignant degeneration and proliferation of transplanted stem cells (including possible genomic or epigenetic changes) in the longer term, and infection (including zoonotic infections with virus integration), as well as potential immune reactions, need to be identified in more stringent clinical trials. Appropriate government regulation of these regenerative therapies is essential given scientific and clinical analysis for the safe development and implementation of the rapidly expanding landscape of local bioactive agents and regenerative cell therapy.

### 3.4. Gene Therapy

Gene therapy involves the replacement or upregulation of specific genes to repair tissue damage and regenerate nerves. At present, gene therapy for ED can be divided into three main components: activators of the nitrergic–neural system, endothelial growth factors (GFs) promoters, and modulators of ion channels in smooth muscle cells [31]. Various growth factors have been explored as gene therapies for ED including brain-derived neurotrophic factor (BDNF) and glial cell-derived neurotrophic factor (GDNF), usually delivered via a vector such as adeno-associated and herpes simplex viruses [63]. While various preclinical investigations have been conducted on gene therapy to treat various causes of ED such as ageing, diabetes and cavernous nerve injury (CNI) animal models with relatively good outcomes, it remains far from reality whether gene therapy can be adopted as the standard of care in humans. So far, the only human phase 1 trial was published more than 15 years ago [64].

While the outcomes of these neuromodulatory GFs as a possible therapeutic option in the management of ED in various animal models have been promising, numerous unanswered concerns remain especially regarding long term outcomes and safety issues such as the risk of an excessive inflammatory response, transgenic infection and carcinogenesis. In addition to the vector delivery system, various tissue promoters and enhancers are required to improve the efficacy of gene therapy and potentially minimize adverse effects. Future studies need to be conducted to examine the efficacy of one GF agent over another, proper dosage of the GF agent, the exact mechanism of delivery and ideal time course for therapy delivery.

### 3.5. Vascular Stent

Coronary artery stents have revolutionized the treatment of men with ischemic heart disease and this minimally invasive endovascular intervention has offered many patients who are frail and not suitable for open cardiac bypass a renewed hope. Given the strong correlation between the presence of angiographic coronary artery and internal pudendal artery disease in men with ED [65], attempts have been made to perform microvascular stenting to treat men with arteriogenic ED. Clinical trials such as the Pelvic Angiography in Non-responders to PDE5 Inhibitors (PANPI trial), the Zotarolimus–Eluting Peripheral stent system for the treatment of erectile dysfunction in males with suboptimal response to PDE5 inhibitors (ZEN trial) and the Incidence of Male Pudendal Artery Stenosis in Suboptimal Erections Study (IMPASSE trial) are designed to evaluate the angiographic patterns of atherosclerosis in erectile-related arteries in men with suspected or known coronary artery disease or peripheral artery disease with the added potential aim of microvascular stenting [31].

A more recent balloon angioplasty study for men with isolated arteriogenic ED reported a reasonable and acceptable sustained clinical success [66]. While penile artery angioplasty is clinically feasible and safe, there is a need for a more durable treatment strategy for penile artery stenotic disease, especially as many of these men will invariably develop a generalized vascular disease in the future. More studies are warranted to define the role of endovascular procedures in this ED subpopulation and potential complications, long term safety and efficacy outcomes will need to be addressed before it can be embraced as the standard of care.

### 3.6. Tissue Engineering and Penile Transplant 

The notion of replacing end organ damage with newly engineered tissue is exciting and reconstruction of normal erectile tissue using autologous cells, derived from the patient’s own body, has far-reaching implications not just in the field of ED but also in other reconstructive cases. While Atala and his group have been at the forefront of tissue engineering [67], many issues remain unresolved including clinical translation to human subjects (especially the safety and efficacy of these engineered materials). Furthermore, the creation of custom-made bioengineered organs using acellular scaffolds and matrices in regenerative medicine is still far from ideal and further research to develop novel biomaterials and cellular sources, coupled with improvements in tissue engineering techniques, will no doubt assist in this “bench-to-bedside” translational research.

On another hand, penile transplantation using vascularized composite allografts is an emerging technique to treat genital loss [68,69]. Penile transplantation allows for restoring both urinary and sexual function by providing a highly functional conduit for urination and a “normal”-appearing and functional organ for sexual intimacy. This complex reconstructive surgery would surpass many of the pitfalls of current penile reconstruction, particularly neophalloplasty. Conventional genital reconstruction is often associated with complications such as suboptimal cosmetic appearance, urethral fistula and/or stricture development, and the inability to restore “normal” erectile function as well as the need for multiple complex procedures [70].

However, the process of selecting the appropriate candidate for genitourinary vascularized composite allograft surgery is rigorous with extensive clinical visits, laboratory testing, imaging and psychological evaluations [71]. Side effects of high dose immunosuppression, including infection and renal toxicity, should be discussed and recipients are screened for possible rejection of the graft with multiple tissue biopsies and regular blood test monitoring for the rest of their life. Pertinent questions such as donor/recipient programs, psychological implications, cost modelling and ethical concerns will warrant further discussion [72].

## 4. Conclusions

There have been considerable scientific advances made in the field of ED in recent years. Existing ED therapies are far from ideal and likely unable to address the growing medical needs of our ageing patient population. Innovative and novel ED therapies currently under development may be able to reverse, regenerate and replace underlying diseased endothelial, neural and penile vascular smooth muscle cells in the very near future. If proven to be safe and effective in the longer-term, these state-of-the-art therapeutic agents will transform the way ED is managed and perhaps cure ED once and for all.

**Table 1:** Current and emerging therapeutic options for erectile dysfunction.

Therapeutic Agents	Mechanism(s) of Action	Additional Description	References
PDE5i	Inhibits PDE5 enzyme to increase cGMP and NO release	1st generation: Sildenafil, Vardenafil, Tadalafil 2nd generation: Avanafil, Mirodenafil, lodenafil Udenafil	[3,4,5,6,7,8,9,10,11,12]
Intracavernosal agents	1. Prostaglandin E1 (Alprostadil) stimulates cAMP release 2. Papaverine is a non-selective PDE5i 3. Phentolamine is a non-selective alpha-adrenergic antagonist 4. Aviptadil is a synthetic VIP	Available as single agent or in combination as Bimix (2 agents), Trimix (3 agents) or Quadmix (including atropine).	[13,14,15,16]
Penile prosthesis implant	1. Non-inflatable (malleable) implant consists of a pair of rods 2. Inflatable implant (usually 3-piece) consists of a reservoir, pair of cylinders and a pump	Boston Scientific AMS 700 series and Coloplast Titan series are the dominant 3-piece inflatable penile prostheses in the market	[22,23,24]
Penile revascularisation surgery	Anastomosis of the inferior epigastric artery to the dorsal penile arteries and/or the deep dorsal vein	Should only be offered to younger non-diabetic men (<55 years) with proven isolated arterial stenosis, usually in the setting of pelvic trauma and without generalized vascular disease	[25,26,27]
Novel drug or drug delivery systems	1. Guanylate cyclase (GC) activators (e.g., BAY 60-2770) 2. RhoA/Rho-kinase inhibitor (e.g., Fasudil)	Drug delivery systems using oral disintegrating formulations and nanotechnology (e.g., nanoethosomes, transfersomes and penetration enhancing lipid vesicles)	[28,29,30,31,32,33,34,35,36,37,38,39,40,41]
Low intensity shockwave therapy	Promotes neovascularization through release of various angiogenic growth factors and activation tissue repair functions (e.g., enhanced macrophage activity, alteration in cellular apoptosis, greater synthesis of cellular proteins and activation as well as enhanced recruitment and subsequent differentiation of stem/progenitor cells)	Various machines, treatment templates and protocols	[42,43,44,45,46]
Cellular-based therapy	1. Stem-cell therapy 2. Platelet-rich plasma therapy	Methods to enhance the therapeutic effects with the use of growth factors, gene manipulation and matrixen	[47,48,49,50,51,52,53,54,55,56,57,58,59,60,61]
Gene therapy	1. Activator of nitrergic–neural system 2. Endothelial GFs promoters 3. Modulator of ion channels in smooth muscle cells	Concurrent use of growth factors and vector delivery system	[31,63,64]
Vascular stents	Provides microvascular stenting and balloon angioplasty	Requires angiographic evaluation for patterns of atherosclerosis in erectile-related arteries in men with suspected or known coronary artery disease or peripheral artery disease	[31,65,66]
Tissue engineering and penile transplant	Tissue engineering using biomaterials, acellular scaffolds and matrices Penile transplant using vascularized composite allografts	Issues relating to cost, intensive programs, immunosuppression, psychological, safety and ethical concerns	[67,68,69,70,71,72]

PDE5—phosphodiesterase type 5; cGMP—cyclic guanosine monophosphate; NO—nitric oxide; cAMP—cyclic adenosine monophosphate; VIP—Vasoactive Intestinal Peptide; GF—growth factor.

## References

[1] Lewis R.W., Fugl-Meyer K.S., Bosch R., Fugl-Meyer A.R., Laumann E.O., Lizza E., Martin-Morales A. (2004). Epidemiology/risk factors of sexual dysfunction. J. Sex Med..

[2] Nehra A., Jackson G., Miner M., Billups K.L., Burnett A.L., Buvat J., Billups K.L., Burnett A.L., Buvat J., Carson C.C. (2012). The Princeton III Consensus recommendations for the management of erectile dysfunction and cardiovascular disease. Mayo Clin. Proc. Mayo Clin..

[3] Hatzimouratidis K., Salonia A., Adaikan G., Buvat J., Carrier S., El-Meliegy A., McCullough A., Torres L.O., Khera M. (2016). Pharmacotherapy for erectile dysfunction: Recommendations from the fourth international consultation for sexual medicine (ICSM 2015). J. Sex Med..

[4] Smith-Harrison L.I., Patel A., Smith R.P. (2016). The devil is in the details: An analysis of the subtleties between phosphodiesterase inhibitors for erectile dysfunction. Transl. Androl. Urol..

[5] Greenberg D.R., Richardson M.T., Tijerina J.D., Bass M.B., Eisenberg M.L. (2019). The Quality of Systematic Reviews and Meta-Analyses in Erectile Dysfunction Treatment and Management Published in the Sexual Medicine Literature. J. Sex Med..

[6] Yuan J., Zhang R., Yang Z., Lee J., Liu Y., Tian J., Qin X., Ren Z., Ding H., Chen Q. (2013). Comparative effectiveness and safety of oral phosphodiesterase type 5 inhibitors for erectile dysfunction: A systematic review and network meta-analysis. Eur. Urol..

[7] Limoncin E., Gravina G.L., Corona G., Maggi M., Ciocca G., Lenzi A., Jannini E.A. (2017). Erectile function recovery in men treated with phosphodiesterase type 5 inhibitor administration after bilateral nerve-sparing radical prostatectomy: A systematic review of placebo-controlled randomized trials with trial sequential analysis. Andrology.

[8] Yang L., Qian S., Liu L., Pu C., Yuan H., Han P., Wei Q. (2013). Phosphodiesterase-5 inhibitors could be efficacious in the treatment of erectile dysfunction after radiotherapy for prostate cancer: A systematic review and meta-analysis. Urol Int..

[9] Vardi M., Nini A. (2007). Phosphodiesterase inhibitors for erectile dysfunction in patients with diabetes mellitus. Cochrane Database Syst. Rev..

[10] Liao X., Qiu S., Bao Y., Wang W., Yang L., Wei Q. (2019). Comparative efficacy and safety of phosphodiesterase type 5 inhibitors for erectile dysfunction in diabetic men: A Bayesian network meta-analysis of randomized controlled trials. World J. Urol..

[11] Jannini E.A., DeRogatis L.R., Chung E., Brock G.B. (2012). How to evaluate the efficacy of the phosphodiesterase type 5 inhibitors. J. Sex Med..

[12] Chen L., Staubli S.E., Schneider M.P., Kessels A.G., Ivic S., Bachmann L.M., Kessler T.M. (2015). Phosphodiesterase 5 inhibitors for the treatment of erectile dysfunction: A trade-off network meta-analysis. Eur. Urol..

[13] Belew D., Klaassen Z., Lewis R.W. (2015). Intracavernosal injection for the diagnosis, evaluation, and treatment of erectile dysfunction: A review. Sex Med. Rev..

[14] Wyllie M.G. (2010). The anatomy of drug development: Invicorp, a product before its time. BJU Int..

[15] Burnett A.L., Nehra A., Breau R.H., Culkin D.J., Faraday M.M., Hakim L.S., Heidelbaugh J., Khera M., McVary K.T., Miner M.M. (2018). Erectile dysfunction: AUA guideline. J. Urol..

[16] El-Sakka A.I. (2016). What is the current role of intracavernosal injection in management of erectile dysfunction. Int. J. Import Res..

[17] Costa P., Potempa A.J. (2012). Intraurethral alprostadil for erectile dysfunction: A review of the literature. Drugs.

[18] Raina R., Agarwal A., Zaramo C.E., Ausmundson S., Mansour D., Zippe C.D. (2005). Long-term efficacy and compliance of MUSE for erectile dysfunction following radical prostatectomy: SHIM (IIEF-5) analysis. Int. J. Impot Res..

[19] Rooney M., Pfister W., Mahoney M., Nelson M., Yeager J., Steidle C. (2009). Long-term, multicenter study of the safety and efficacy of topical alprostadil cream in male patients with erectile dysfunction. J. Sex Med..

[20] Cai T., Palumbo F., Liguori G., Mondaini N., Scroppo F.I., Di Trapani D., Cocci A., Zucchi A., Verze P., Salonia A. (2019). The intra-meatal application of alprostadil cream (Vitaros®) improves drug efficacy and patient’s satisfaction: Results from a randomized, two-administration route, cross-over clinical trial. Int. J. Impot Res..

[21] Scott F.B., Bradley W.E., Timm G.W. (1973). Management of erectile impotence: Use of implantable inflatable prosthesis. Urology.

[22] Chung E. (2017). Penile prosthesis implant: Scientific advances and technological innovations over the last four decades. Transl. Androl. Urol..

[23] Chung E. (2017). Translating penile erectile hydraulics to clinical application in inflatable penile prosthesis implant. Curr. Sex. Health Rep..

[24] Pastuszak A.W., Lentz A.C., Farooq A., Jones L., Bella A.J. (2015). Technological improvements in three-piece inflatable penile prosthesis design over the past 40 years. J. Sex Med..

[25] Michal V., Kramar R., Pospichal J. (1974). Femoro-pudendal bypass, internal iliac thromboendarterectomy and direct arterial anastomosis to the cavernous body in the treatment of erectile impotence. Bull. Soc. Int. Chir..

[26] Virag R., Bennett A.H. (1982). Revascularization of the Penis. Management of Male Impotence.

[27] Trost L.W., Munarriz R., Wang R., Morey A., Levine L. (2016). External mechanical devices and vascular surgery for erectile dysfunction. J. Sex Med..

[28] Stasch J.P., Schmidt P.M., Nedvetsky P.I., Nedvetskaya T.Y., Arun Kumar H.S., Meurer S. (2006). Targeting the heme-oxidized nitric oxide receptor for selective vasodilatation of diseased blood vessels. J. Clin. Investig..

[29] Lasker G.F., Pankey E.A., Frink T.J., Zeitzer J.R., Walter K.A., Kadowitz P.J. (2013). The sGC activator BAY 60-2770 has potent erectile activity in the rat. Am. J. Physiol. Heart Circ. Physiol..

[30] Estancial C.S., Rodrigues R.L., De Nucci G., Antunes E., Mónica F.Z. (2015). Pharmacological characterisation of the relaxation induced by the soluble guanylate cyclase activator, BAY 60-2770 in rabbit corpus cavernosum. BJU Int..

[31] Chung E., Brock G.B. (2011). Emerging and novel therapeutic approaches in the treatment of male erectile dysfunction. Curr. Urol. Rep..

[32] Jin L., Burnett A.L. (2006). RhoA/Rho-kinase in erectile tissue: Mechanisms of disease and therapeutic insights. Clin. Sci..

[33] Park K., Kim S.W., Rhu K.S., Paick J.S. (2006). Chronic administration of an oral Rho-kinase inhibitor prevents the development of vasculogenic erectile dysfunction in a rat model. J. Sex Med..

[34] Guagnini F., Ferazzini M., Grazzo M., Blanco S., Croci T. (2012). Erectile properties of the Rho-kinase inhibitor SAR407899 in diabetic animals and human isolated corpora cavernosa. J. Transl. Med..

[35] Goel H., Rai P., Rana V. (2008). Orally disintegrating systems: Innovations in formulation and technology. Recent Pat. Drug Deliv. Formul..

[36] Cocci A., Capece M., Cito G., Russo G.I., Falcone M., Timpano M., Rizzo M., Della Camera P.A., Morselli S., Campi R. (2017). Effectiveness and Safety of Oro-Dispersible Sildenafil in a New Film Formulation for the Treatment of Erectile Dysfunction: Comparison Between Sildenafil 100-mg Film-Coated Tablet and 75-mg Oro-Dispersible Film. J. Sex Med..

[37] Chung E., Brock G.B. (2011). A state of art review on vardenafil and erectile dysfunction. Expert Opin. Pharmother..

[38] Kurakula M., Ahmed O.A., Fahmy U.A., Ahmed T.A. (2016). Solid lipid nanoparticles for transdermal delivery of avanafil: Optimization, formulation, in-vitro and ex-vivo studies. J. Liposome Res..

[39] Sala M., Diab R., Elaissari A., Fessi H. (2018). Lipid nanocarriers as skin drug delivery systems: Properties, mechanisms of skin interactions and medical applications. Int. J. Pharm..

[40] Fahmy U.A. (2015). Nanoethosomal transdermal delivery of vardenafil for treatment of erectile dysfunction: Optimization, characterization, and in vivo evaluation. Drug Des. Devel. Ther..

[41] Berkó S., Zsikó S., Deák G., Gácsi A., Kovács A., Budai-Szűcs M., Pajor L., Bajory Z., Csányi E. (2018). Papaverine hydrochloride containing nanostructured lyotropic liquid crystal formulation as a potential drug delivery system for the treatment of erectile dysfunction. Drug Des. Devel. Ther..

[42] Xu L., Zhao Y., Wang M., Song W., Li B., Liu W., Jin X., Zhang H. (2016). Defocused low-energy shock wave activates adipose tissue-derived stem cells in vitro via multiple signaling pathways. Cytotherapy.

[43] Ito K., Fukumoto Y., Shimokawa H. (2009). Extracorporeal shock wave therapy as a new and non-invasive angiogenic strategy. Tokohu J. Exp. Med..

[44] Clavijo R.I., Kohn T.P., Kohn J.R., Ramasamy R. (2017). Effects of low-intensity extracorporeal shockwave therapy on erectile dysfunction: A systematic review and meta-analysis. J. Sex Med..

[45] Lu Z., Lin G., Reed-Maldonado A., Wang C., Lee Y.C., Lue T.F. (2017). Low intensity extracorporeal shockwave therapy improves erectile dysfunction: A systematic review and meta-analysis. Eur. Urol..

[46] Chung E., Wang J. (2017). A state-of-art review of low intensity extracorporeal shock wave therapy and lithotripter machines for the treatment of erectile dysfunction. Expert. Rev. Med. Devices..

[47] Chung E. (2015). Stem-cell-based therapy in the file of urology: A review of stem cell basic science, clinical applications and future directions in the treatment of various sexual and urinary conditions. Expert. Opin. Biol. Ther..

[48] Chung E. (2019). Stem cell therapy in diabetic men with erectile dysfunction: A step closer to safe and effective regenerative technology. Ann. Transl. Med..

[49] Lin C.S., Xin Z., Dai J., Huang Y.C., Lue T.F. (2013). Stem-cell based therapy for erectile dysfunction. Expert. Opin. Biol. Ther..

[50] Qiu X., Sun C., Yu W., Lin H., Sun Z., Chen Y., Wang R., Dai Y. (2012). Combined strategy of mesenchymal stem cell injection with vascular endothelial growth factor gene therapy for treatment of diabetes-associated erectile dysfunction. J. Androl..

[51] He Y., He W., Qin G., Luo J., Xiao M. (2013). Transplantation KCNMA1 modified bone marrow mesenchymal stem cell therapy for diabetes mellitus-induced erectile dysfunction. Andrologica.

[52] Kim S.J., Park S.H., Sung Y.C., Kim S.W. (2012). Effect of mesenchymal stem cells associated to matrixen on the erectile function in the rat model with bilateral cavernous nerve crushing injury. Int. Braz. J. Urol..

[53] Yiou R., Hamidou L., Birebent B., Bitari D., Lecorvoisier P., Contremoulins I., Khodari M., Rodriguez A.M., Augustin D., Roudot-Thoraval F. (2016). Safety of Intracavernous Bone Marrow-Mononuclear Cells for Postradical Prostatectomy Erectile Dysfunction: An Open Dose-Escalation Pilot Study. Eur. Urol..

[54] Al Demour S., Jafar H., Adwan S., AlSharif A., Alhawari H., Alrabadi A., Zayed A., Jaradat A., Awidi A. (2018). Safety and Potential Therapeutic Effect of Two Intracavernous Autologous Bone Marrow Derived Mesenchymal Stem Cells injections in Diabetic Patients with Erectile Dysfunction: An Open Label Phase I Clinical Trial. Urol Int..

[55] Yang J., Yu Z., Zhang Y., Zang G.H., Zhuan L., Tang Z., Liu Y., Wang T., Wang S.G., Liu J.H. (2019). Preconditioning of adipose-derived stem cells by phosphodiesterase-5 inhibition enhances therapeutic efficacy against diabetes-induced erectile dysfunction. Andrology.

[56] Wu Y.N., Wu C.C., Sheu M.T., Chen K.C., Ho H.O., Chiang H.S. (2016). Optimization of platelet-rich plasma and its effects on the recovery of erectile function after bilateral cavernous nerve injury in a rat model. J. Tissue Eng. Regen. Med..

[57] El-Sharkawy H., Kantarci A., Deady J., Hasturk H., Liu H., Alshahat M., Van Dyke T.E. (2007). Platelet rich plasma: Growth factors and pro- and anti-inflammatory properties. J. Periodontol..

[58] Epifanova M.V., Chalyi M.E., Krasnov A.O. (2017). Investigation of mechanisms of action of growth factors of autologous factors platelet-rich plasma used to treat erectile dysfunction. Urologiia.

[59] Matz E.L., Pearlman A.M., Terlecki R.P. (2018). Safety and feasibility of platelet rich fibrin matrix injections for treatment of common urologic conditions. Invest. Clin. Urol..

[60] Chahla J., Cinque M.E., Piuzzi N.S., Mannava S., Geeslin A.G., Murray I.R., Dornan G.J., Muschler G.F., LaPrade R.F. (2017). A call for standardization in platelet-rich Plasma preparation protocols and composition reporting: A systematic review of the clinical orthopaedic literature. J. Bone Joint Surg Am..

[61] Scott S., Roberts M., Chung E. (2019). Platelet-rich plasma and treatment of erectile dysfunction: Critical review of literature and global trends in platelet-rich plasma clinics. Sex Med. Rev..

[62] Protogerou V., Mihalopoulos E., Mallis P., Gontika I., Dimou Z., Liakouras C., Stavropoulos-Giokas C., Kostakopoulos N., Chrisofos M., Deliveliotis C. (2019). Administration of Adipose Derived Mesenchymal Stem Cells and Platelet Lysate in Erectile Dysfunction: A Single Center Pilot Study. Bioengineering.

[63] Yoshimura N., Kato R., Chancellor M.B., Nelson J.B., Glorioso J.C. (2010). Gene therapy as future treatment of erectile dysfunction. Expert Opin. Biol. Ther..

[64] Melman A., Bar-Charma N., McCullough A., Davies K., Christ G. (2005). The first human trial of gene transder therapy for the treatment of erectile dysfunction: Preliminary results. Eur. Urol..

[65] Rogers J.H., Karimi H., Kao J., Link D., Javidan J., Yamasaki D.S., Dolan M., Laird J.R., Low R.I. (2010). Internal pudendal artery stenoses and erectile dysfunction: Correlation with angiographic coronary artery disease. Catheter Cardiovasc Interv..

[66] Wang T.D., Lee W.J., Yang S.C., Lin P.C., Tai H.C., Liu S.P., Huang C.H., Chen W.J., Chen M.F., Hsieh J.T. (2016). Clinical and Imaging Outcomes up to 1 Year Following Balloon Angioplasty for Isolated Penile Artery Stenoses in Patients With Erectile Dysfunction: The PERFECT-2 Study. J. Endovac Ther..

[67] Patel M.N., Atala A. (2011). Tissue engineering of the penis. Sci. World J..

[68] Hu W., Lu J., Zhang L. (2006). A preliminary report of penile transplantation. Eur. Urol..

[69] Hu W., Lu J., Zhang L. (2006). A preliminary report of penile transplantation: Part 2. Eur. Urol..

[70] Sopko N.A., Tuffaha S., Lough D., Brandacher G., Lee W.P.A., Bivalacqua T.J., Redett R.J., Burnett A.L. (2017). Penile allotransplantation for complex genitourinary reconstruction. J. Urol..

[71] Szafran A.A., Redett R., Burnett A.L. (2018). Penile transplantation: The US experience and institutional program set-up. Transl. Androl. Urol..

[72] (2016). Modern Medicine Feature Articles Penile transplant: Procedure raises technical, ethical issues. Urology Times.

